# Entrustable professional activity 7: opportunities to collaborate on evidence-based medicine teaching and assessment of medical students

**DOI:** 10.1186/s12909-019-1764-y

**Published:** 2019-09-03

**Authors:** Joey Nicholson, Judy M. Spak, Iris Kovar-Gough, Elizabeth R. Lorbeer, Nancy E. Adams

**Affiliations:** 1 NYU Health Sciences Library, NYU School of Medicine, 550 First Avenue, New York, NY 10016 USA; 2 Harvey Cushing/John Hay Whitney Medical Library, Yale University, New Haven, CT USA; 3 Michigan State University Libraries, Michigan State University, East Lansing, MI USA; 4 Department of the Medical Library, Western Michigan University, Homer Stryker M.D. School of Medicine, Kalamazoo, MI USA; 5 Harrell Health Sciences Library, Penn State College of Medicine, Hershey, PA USA

**Keywords:** Evidence-based medicine, Entrustable professional activities (EPAs), Librarians, Assessment, Curriculum development, Interprofessional

## Abstract

**Background:**

This study was conducted to examine gaps and opportunities for involvement of librarians in medical education and patient care as well as improve the teaching and assessment of Entrustable Professional Activity 7 (EPA 7) -- the ability to form clinical questions and retrieve evidence to advance patient care.

**Methods:**

The Association of Academic Health Sciences Libraries (AAHSL) Competency-Based Medical Education Task Force surveyed all AAHSL member libraries in October 2016 on health sciences librarian awareness and involvement in teaching and assessing EPA 7.

**Results:**

The survey response rate was 54% (88/164 member libraries). While 90% (*n* = 76) of respondents were regularly engaged in teaching or assessing aspects of EPA 7 only 34 (39%) were involved explicitly in a Core EPA 7 project, 44% (15/34) of these projects were librarian initiated.

**Conclusions:**

Involvement in teaching and assessment of EPA 7 is an untapped opportunity for librarians to collaborate in medical education and patient care. Although librarians are already deeply involved in teaching and assessment of EPA 7 related knowledge, skills, and behaviors, further librarian collaboration can help bolster the planning or updating of existing curricula and assessments of this entrustable professional activity.

**Electronic supplementary material:**

The online version of this article (10.1186/s12909-019-1764-y) contains supplementary material, which is available to authorized users.

## Background

The Association of American Medical Colleges (AAMC) introduced guidelines for Entrustable Professional Activities (EPAs) in 2014 as a way to operationalize competency-based medical education by defining core professional tasks that every medical student could be expected to perform independently upon graduation [1]. The AAMC’s Core EPAs describe thirteen discrete tasks, ranging from gathering the patient history and performing a physical examination to identifying system failures. The EPA framework is being adopted at medical schools across the United States, including ten pilot institutions. Early data show that new residents’ performance in the AAMC’s Core EPAs is of variable quality, with residency program directors reporting poor performance in several of the 13 professional activities including EPA 7 [2, 3].

EPA 7, the ability to form clinical questions and retrieve evidence to advance patient care, is a foundational EPA integral to the practice of evidence-based medicine (EBM). In addition to improving patient care, the ability to formulate questions and locate evidence is an essential skill for meeting lifelong learning goals [4, 5]. Yet, the teaching and assessment of EPA 7 may be suboptimal for several reasons including: “sub-optimal role models, student lack of willingness to admit uncertainty, lack of clinical context, and difficulty mastering EBM skills” [6]. While the ability of trainees to demonstrate competence in EPA 7 is an expectation, the same cannot be said of medical schools’ ability to provide comprehensive training and assessment of this EPA [6, 7].

Academic health sciences librarians have a history of teaching and assessing these tasks as part of EBM training and are thus well-positioned to partner with other medical educators in addressing this EPA [8, 9]. To better understand the teaching and assessment of EPA 7 by health sciences librarians and help guide curriculum reform, we conducted a survey to assess the current level of engagement of US and Canadian health sciences librarians and highlight gaps and opportunities for improvement in teaching and assessing EPA 7: *Form clinical questions and retrieve evidence to advance patient care*.

## Methods

A task force of eleven academic health sciences librarians working in medical education curricula across all levels (undergraduate, graduate, and continuing) developed a survey, based on a previously published instrument [10] and informed by qualitative interviews with librarians and medical educators at two of the medical schools serving as pilot institutions for the AAMC’s EPA project. The Wilder Collaboration Factors Inventory also informed the survey questions [11]. Most questions on the survey allowed respondents to add qualitative comments. The survey was administered using Qualtrics™ hosted by Penn State University. After pilot-testing with a small group of health sciences librarians, the link to the survey was electronically distributed to directors of member libraries of the Association of Academic Health Sciences Libraries (AAHSL) (*N* = 164) via the AAHSL discussion list. The President of AAHSL asked the directors to select the librarian who was most familiar with the library’s curricular involvement in the undergraduate medical education program to complete the survey on behalf of the library between October 7 and October 28, 2016. The Michigan State University Institutional Review Board ruled that this survey was exempt from human subjects protection (IRB #× 16-1165e; i052241). The survey is included in Additional file 1.

Survey responses were downloaded to Excel™. Data were cleaned by removing duplicate responses from the same institution and incomplete responses. Excel™ was used to calculate descriptive statistics. Qualitative data was examined to select representative quotes to support the descriptive statistics.

## Results

Of the 164 member libraries, 88 institutions responded to the survey, a response rate of 54% which is an acceptable response rate for an electronic survey [12]. The survey contained branching items, and not all respondents replied to all questions, which accounts for varying response rates in the results. Table 1 provides a summary of the publicly available characteristics of responding institutions, reflecting the diversity of academic health sciences library types, sizes, librarian faculty status, and student class sizes.

**Table 1:** Publicly Available Characteristics of Responder Institutions (*n* = 88), Association of Academic Health Sciences Libraries Annual Statistics 2016

Characteristic	Public Universities (n = 52)	Private Universities (*n* = 36)
Number of Librarians (%)		
0–10	24 (36)	14 (39)
11–20	18 (45)	19 (53)
21–30	3 (6)	2 (5)
Unknown	7 (13)	1 (3)
Librarian Faculty Status (%)		
Yes	36 (69)	6 (17)
No	6 (11)	19 (53)
Equivalent	1 (2)	6 (17)
Some, Not All	4 (8)	5 (13)
Other*	5 (10)	0 (0)
Average Class Size	162.24	152

*Other includes staff or faculty associate status or those transitioning to faculty status

### How involved are libraries in EPAs generally, and EPA 7 in particular?

Of the 88 respondents, 33 (38%) stated that their library was working with their medical school to implement Core EPAs in the undergraduate medical curriculum and an additional two libraries responded that they were independently working on a Core EPA project. A greater number of respondents (*n* = 53, 60%), however, were either: unsure whether Core EPAs were being implemented at the institution at all, not involved in existing EPA projects, or at institutions in which no EPA work was being done.

Of those libraries who were involved in working on Core EPA focused projects (*n* = 35, 40%), libraries were most frequently engaged in projects addressing EPA 7: Form clinical questions and retrieve evidence to advance patient care (*n* = 34, 63%) and EPA 9: Collaborate as a member of an interprofessional team (*n* = 20, 37%) and were involved in other EPAs to a lesser extent. All respondents were asked if they taught or assessed the functions of EPA 7 as standard practice, regardless of whether their institution was implementing Core EPA work, and 84 libraries answered this question. Most libraries, 90% (*n* = 76) of responding institutions indicated that the library was involved in teaching or assessing component functions of EPA 7 as part of their standard practice. Furthermore, when comparing Core EPA project-implementing libraries to those not explicitly involved in Core EPA projects, Pearson Chi Square analyses showed that there were no statistically significant differences in involvement in teaching or assessing any of the eight component functions of EPA 7 between these two groups of libraries (see Table 2).

**Table 2:** Association Between Libraries’ Involvement in Core EPA Projects and Teaching and Assessing of EPA 7 Component Tasks (*n* = 88)

Component Tasks of EPA 7	Teaching	Assessing
Develop a well-formed, focused, pertinent clinical question	X^2^ (3)> = 2.904, *p* = 0.407	X^2^ (3)> = 4.240, *p* = 0.237
Demonstrate basic awareness and early skills in appraisal of both the sources and content of medical information using accepted criteria	X^2^ (3)> = 3.392, *p* = 0.335	X^2^ (3)> = 2.780, *p* = 0.427
Identify and demonstrate the use of information technology to access accurate and reliable online medical information	X^2^ (3)> = 5.084, *p* = 0.166	X^2^ (3)> = 3.581, *p* = 0.310
Demonstrate basic awareness and early skills in assessing applicability/generalizability of evidence and published studies to specific patients	X^2^ (3)> = 1.676, *p* = 0.642	X^2^ (3)> = 2.226, *p* = 0.527
Demonstrate curiosity, objectivity, and the use of scientific reasoning in acquisition of knowledge and application to patient care	X^2^ (3)> = 7.163, *p* = 0.067	X^2^ (3)> = 4.139, *p* = 0.247
Apply the primary findings of one’s information search to an individual patient(s)	X^2^ (3)> = 4.634, *p* = 0.201	X^2^ (3)> = 6.611, *p* = 0.085
Communicate one’s findings to the health care team (including the patient/family)	X^2^ (3)> = 4.677, *p* = 0.197	X^2^ (3)> = 6.429, *p* = 0.092
Close the loop through reflection on the process and the outcome for the patient	X^2^ (3)> = 3.923, *p* = 0.270	X^2^ (3)> = 2.195, *p* = 0.533

### Which EPA 7 tasks are being taught and assessed?

EPA 7 consists of eight component tasks, results indicated that libraries were primarily involved in teaching the first five tasks of EPA 7 (teaching > 50%): forming a clinical question, using information technology to access medical information, appraising sources and content of medical information using accepted criteria, demonstrating skill in assessing applicability of evidence to patients, and demonstrating curiosity and the use of scientific reasoning in acquiring knowledge and applying to care. In terms of assessment, libraries were primarily involved in assessing the first three tasks of EPA 7 (assessing > 50%). Libraries reported much lower involvement in assessment across all component tasks. The EPA 7 task least taught and assessed by librarians was “closing the loop through reflection on the process and the outcome for the patient.” For each of the eight component tasks of EPA 7, libraries were teaching these skills more than assessing them. For example, the task “Identify and demonstrate the use of information technology to access accurate and reliable online medical information” was the EPA 7 component most frequently addressed by academic health sciences libraries in the medical curriculum. Of responding libraries, 87% (*n* = 74) reported that the library was involved in teaching this component of EPA 7 while only 61% of responding libraries (*n* = 52) reported that they assessed this component of EPA 7 (see Table 3).

**Table 3:** Libraries’ Teaching and Assessing of EPA 7 Component Tasks (*n* = 85)

Component Tasks of EPA 7	Teaching n (%)	Assessing n (%)	Difference in Involvement
Develop a well-formed, focused, pertinent clinical question	72 (85%)	49 (58%)	−27%
Demonstrate basic awareness and early skills in appraisal of both the sources and content of medical information using accepted criteria	68 (80%)	47 (55%)	− 25%
Identify and demonstrate the use of information technology to access accurate and reliable online medical information	74 (87%)	52 (61%)	−26%
Demonstrate basic awareness and early skills in assessing applicability/generalizability of evidence and published studies to specific patients	54 (64%)	35 (41%)	−23%
Demonstrate curiosity, objectivity, and the use of scientific reasoning in acquisition of knowledge and application to patient care	45 (54%)	22 (26%)	−28%
Apply the primary findings of one’s information search to an individual patient(s)	41 (48%)	27 (32%)	−16%
Communicate one’s findings to the health care team (including the patient/family)	29 (34%)	21 (25%)	−9%
Close the loop through reflection on the process and the outcome for the patient	24 (28%)	13 (15%)	−13%

a Not all respondents answered each item, all percentages are calculated based on 85 total possible respondents.

### In which stages of the curriculum are EPA 7 tasks taught and assessed?

We also probed in what stage of the curriculum a library was involved in teaching and assessing component tasks of EPA 7. For each of the component tasks of EPA 7, respondents were asked whether their library was involved in the preclinical curriculum, clinical curriculum, both portions, or not at all. For the first five EPA 7 tasks, where libraries report being the most involved, teaching in both preclinical and clinical stages of the curriculum ranged from 22 to 42% (task-dependent). However, teaching in the preclinical stage alone ranged from 16 to 27% of respondents. For these same five EPA 7 tasks, 11–20% of libraries report assessing them in both stages of the curriculum whereas 5–25% of libraries reported assessing them in the preclinical phase only (see Table 4).

**Table 4:** Location in Curriculum of EPA 7 Teaching and Assessment Activities by Librarians (*n* = 85)

	Teaching n (%)					Assessment n (%)				
EPA 7 Component Tasks	PC	C	B	N	NR	PC	C	B	N	NR
Develop a well-formed, focused, pertinent clinical question	27 (32)	11 (13)	34 (40)	13 (15)	–	20 (24)	11 (13)	18 (21)	34 (40)	2 (2)
Demonstrate basic awareness and early skills in appraisal of both the sources and content of medical information using accepted criteria	26 (30)	10 (12)	32 (38)	17 (20)	–	21 (25)	9 (11)	17 (20)	36 (42)	2 (2)
Identify and demonstrate the use of information technology to access accurate and reliable online medical information	27 (32)	5 (6)	42 (49)	11 (13)	–	25 (29)	7 (8)	20 (24)	30 (35)	3 (3)
Demonstrate basic awareness and early skills in assessing applicability/generalizability of evidence and published studies to specific patients	20 (24)	12 (14)	22 (26)	31 (26)	–	16 (19)	8 (9)	11 (13)	47 (55)	3 (3)
Demonstrate curiosity, objectivity, and the use of scientific reasoning in acquisition of knowledge and application to patient care	16 (19)	7 (8)	22 (26)	39 (46)	1 (1)	5 (6)	6 (7)	11 (13)	61 (72)	2 (2)
Apply the primary findings of one’s information search to an individual patient(s)	14 (16)	12 (14)	15 (18)	43 (50)	1 (1)	8 (9)	9 (11)	9 (11)	56 (66)	3 (3)
Communicate one’s findings to the health care team (including the patient/family)	8 (9)	13 (15)	8 (9)	54 (63)	2 (2)	6 (7)	8 (9)	7 (8)	62 (73)	2 (2)
Close the loop through reflection on the process and the outcome for the patient	6 (7)	10 (12)	8 (9)	59 (69)	2 (2)	1 (1)	6 (7)	6 (7)	70 (82)	2 (2)

Abbreviations: *PC* Pre-Clinical, *C* Clinical, *B* Both, *N* Not at All, *NR* No Response

### EPA implementation project involvement

The 34 respondent libraries that indicated involvement in an EPA implementation project in undergraduate medical curricula were further asked to define how the involvement was initiated, funded, and led, and how information about the project was shared between librarians and the medical school faculty. Nearly half of the libraries responding to the question (*n* = 15, 44%) reported that librarians initiated the EPA-related curricular partnerships. The leadership of the EPA project was almost equally spread between: shared leadership among librarians and medical school teaching faculty through library representation on education committees (*n* = 16, 47%) and medical school faculty leadership alone (*n* = 14, 41%), with some respondents reporting unique situations (*n* = 4, 12%). The majority of libraries responding (*n* = 27, 79%) indicated that there was no external funding for their participation in EPA activities and no line item for the project in the library’s budget. When asked how information about the EPA projects were shared between the medical school faculty and librarians, responding libraries were almost evenly split between collaboration, defined as information sharing occurs regularly and leads to long-term projects between medical school faculty and librarians beyond individual class sessions (*n* = 13, 38%) and coordination, in which information is shared pertaining only to the specific needs or tasks related to an instruction session (*n* = 14, 41%), with several respondents indicating information sharing was limited or unkown (*n* = 7, 21%).

The respondents who said that they were involved in EPA related projects were asked about the division of labor for teaching and assessment. Of the 34 possible respondents to this question, 76% (*n* = 26) stated that medical school faculty and librarians were jointly responsible for teaching skills related to the EPAs. Regarding responsibility for assessment, 41% (n = 14) described assessment, including planning and grading, as a joint responsibility of medical school faculty and librarians. Eight participants (24%) were not involved in curricular assessment, indicating that it was the sole responsibility of the medical school faculty.

### Barriers and challenges to EPA involvement

All libraries submitting the survey were asked to rank the significance of various barriers or challenges that may have existed relative to librarians’ involvement in implementation of Core EPA projects on a four-point scale (extremely significant, more significant, less significant, or not significant). The greatest barrier to librarians implementing Core EPA projects was lack of time in the curriculum with 61 libraries (72%) responding that this challenge was either extremely or more significant. Lack of resources such as staff time or funding and a lack of existing useful models of other libraries doing EPA work were also mentioned by approximately half of the respondents as either extremely or more significant barriers. On the other hand, lack of evidence of the value of Core EPAs, “push-back” about EPA concepts by students or faculty, and lack of librarian and/or staff training or expertise in Core EPA-related content were seen as less significant or not significant barriers by the majority of responding libraries.

### Change in curriculum involvement

The 34 respondents who indicated that their libraries were involved in the implementation of Core EPAs at their medical schools were asked to describe how the library’s involvement in the medical curriculum had changed since the implementation of the Core EPAs. Individuals at 28 libraries responded to this question, many of them stating that involvement in Core EPA work had led to increased integration within the curriculum. One respondent indicated that the integration had come about as a result of a shared purpose and goals provided by the EPAs. Another respondent stated that EPA implementation increased librarian involvement in assessment. Some respondents also reported that involvement in Core EPA work generally raised awareness of and respect for the librarians as key partners in medical education. On the other hand, this was not a universal finding. Several respondents (*n* = 12; 35%) said that there have been little or no changes in the library’s involvement as a result of Core EPA implementation (see Table 5).

**Table 5:** Qualitative Comments Regarding Involvement in Curriculum

Increased Involvement in Curriculum	
“We have more dedicated time in the curriculum that we did not have before.”	
“The librarians are more involved in conducting classes for clinical and pre-clinical courses.”	
“There was library involvement in the curriculum prior to the Core EPAs; however, the EPAs have definitely provided a focus for our efforts and a better set of shared language and goals.”	
“…allows us to […] become more involved in assessment. Assessing skills and knowledge for EPAs will require multiple points of data for each EPA and will probably require more performance and portfolio-based assessments.”	
“…has increased the awareness of the value of the library among teaching faculty, lent credibility to librarians’ work because of the mandating of information literacy skills at a higher level, and deepened collaboration and increased partnerships with medical school faculty.”	
Little or No Change in Involvement in Curriculum	
“Our involvement is essentially the same, as are the sessions we teach - only the specific classification of these sessions has changed.”	
“Involvement has evolved over time, independent of the Core EPAs.”	
“I guess a lot of it really is more of the usual information literacy instruction, but we are using it to satisfy some of the EPA requirements.”	

## Discussion

While EPA 7 is directly related to librarians’ expertise, librarians’ access to learners for the purposes of teaching and assessing these skills across the curriculum varied. Our study highlights the vast amount of work being done by librarians to teach and assess EPA 7. However, this work was often unrecognized by curriculum leaders as a formal part of the curriculum and was not captured by existing assessment structures and systems.

Based on our findings, librarians were already regularly teaching five of the eight core tasks of EPA 7, although for most it was not directly tied to an EPA 7 project or implementation. Additionally, these curricular pieces often occured in isolation where librarians only taught the pieces they were asked to teach and were often unaware of how it would connect to the other sessions taught in the EBM curriculum. The first five tasks of EPA 7 fall within the expertise of medical librarians, while the last three tasks may be better suited to collaboration with clinician faculty members. If a learning event is not integrated with other components in the curriculum, then student competency acquisition related to EPA 7 may not be adequately scaffolded and supported. To enhance clarity, curricula on evidence-based medicine that are taught and led by librarians should be explicitly labeled as being part of EPA 7 or an EBM competency. Course and curriculum leaders should also be encouraged to involve librarians in the planning and structure of EBM courses. Possibly more troubling is at what times in the curriculum librarians report being involved in EBM training. While EPA 7 is a clinical expectation, over 50% of librarians’ reported teaching of the content occurs only in the preclinical years, before learners are expected to practice EBM. This may work well for some institutions, but with an eye to the future, EBM education leaders should consider incorporating more librarian training in the clinical years.

Our study also identified a lack of librarian involvement in assessment of EPA 7 skills, with only 25% of respondents reporting being involved in assessing the first three EPA 7 tasks that they are teaching. Assessment that was being conducted by librarians occured mostly in the pre-clinical years, with very little assessment of select steps of EPA 7 taking place in the clinical years. This provides an opportunity to involve librarians longitudinally throughout the curriculum utilizing the EPA framework. Since assessment drives learning, if librarians are involved in teaching these skills, they should also be contributing evidence of EPA 7 attainment from student assessments. Librarians are well-equipped to both be key assessors of student performance and to help in the development and use of existing assessment instruments for this concept.

The barriers of lack of time in the curriculum, lack of resources, and lack of staff time which were reported by respondents are not unique to EBM training. Librarians and curricular leaders should begin to think outside the box and consider innovative models of teaching content longitudinally. Librarians often wear many hats at their institutions and have many competing demands for their time as do medical school faculty and curricular directors. Working closely as collaborators and engaging with librarians as instructors for EBM content could be a way to share responsibilities and scale this engagement effectively.

Much of librarians’ everyday practice in academic health sciences libraries already supports EPA 7—perhaps even without realizing it. If an institution has adopted the EPA approach, then librarians should be utilized as content experts in EPA 7. Since program directors cite EPA 7 as one of the areas in which their residents are least prepared [2, 3], librarians and faculty colleagues should explore additional collaborations to strengthen student learning in this area.

While many librarians were involved in teaching of EPA 7 tasks via regular teaching of evidence-based medicine skills, future research should focus on two different areas: 1) evaluating programming to raise faculty awareness of the benefits of collaboration with librarian colleagues in order to enhance longitudinal EBM teaching and assessment within curricula; and 2) developing better assessment methods and assessment training for librarians to begin using with their instruction to address the assessment gap.

### Limitations

The views expressed in this study were only of librarians from academic health sciences libraries in the U.S. and Canada, and not hospital libraries. Future research should assess the opinions of hospital librarians who may be teaching and assessing EPA 7 clinically as students rotate through their settings. Second, the study authors also contributed their own experiences to the survey data. While this was de-identified for analysis, the authors have extensive experiences working on the Core EPA projects at their own institutions and therefore participated in the survey as respondents representing their libraries. Eliminating this data would have been a misrepresentation of what is being done in academic health sciences libraries in the U.S. and Canada. Nonetheless, the authors feel the data presented here is generalizable to the whole population of academic health sciences libraries. Additionally, we only examined competency in EBM in the undergraduate medical education context, specifically EPA 7. While teaching to and assessing competency in EBM crosses into graduate medical education, that was beyond the scope of this research. Future research should explore how this competency is taught and assessed during the transition to graduate medical education and throughout the continuum in a developmentally appropriate way.

## Conclusions

This study provides insight into gaps and opportunities in teaching and assessment of EPA 7. While much work is being done both to teach and assess EPA 7, institutions could think more strategically about when, how often, and for what purposes to utilize librarians in these efforts. Librarians are already doing this work and should be called upon as partners in development of curricula, teaching, and assessment of EPA 7 in medical students.

## Additional file

Additional file 1: AAHSL EPA survey. (PDF 189 kb)

## Data Availability

The datasets used and/or analysed during the current study are available from the corresponding author on reasonable request.

## Abbreviations

AAHSL: Association of Academic Health Sciences Libraries
AAMC: Association of American Medical Colleges
EBM: Evidence-Based Medicine
EPA: Entrustable Professional Activity
