# Supplementary material for: Entrustable professional activity 7: opportunities to collaborate on evidence-based medicine teaching and assessment of medical students
Source: BMC Med Educ. 2019 Sep 3;19:330. doi: 10.1186/s12909-019-1764-y (PMC6724374; doi:10.1186/s12909-019-1764-y)
Supplement: Supplementary file 1 — AAHSL EPA survey. (PDF 189 kb) [file 12909_2019_1764_MOESM1_ESM.pdf]

AAHSL EPA survey

Q1 Please direct this survey to an individual at the library who can best describe the library's integration into the medical curriculum.
WHO WE ARE The Task Force on Competency-Based Medical Education was formed by the Association of Academic Health Sciences Libraries in March 2016. The purpose of the task force is to identify and communicate the extent to which libraries are participating in changing medical education curricula.
EXPLANATION OF THE RESEARCH and WHAT YOU WILL DO: You are being asked to participate in a research study about competency--based medical education. You will be asked a series of questions about how your library and your medical school are currently involved in competency--based medical education. The survey will take approximately 25-30 minutes to complete. You must be at least 18 years old to participate in this research.
CONTENT OF THE SURVEY In addition to survey questions regarding your library's involvement in competency-based medical education, we will collect your institution's name and your library's name. This will allow us to more fully understand the extent of the work being done by libraries. You may remain anonymous or share your name and contact information in case we have follow-up questions. Personal names will not be published or referenced. Comments will be reported anonymously or in aggregate, unless permission is given.
YOUR RIGHTS TO PARTICIPATE, SAY NO, OR WITHDRAW: Participation in this research project is completely voluntary. You have the right to say no. You may change your mind at any time and withdraw. You may choose not to answer specific questions or to stop participating at any time.
COSTS AND COMPENSATION FOR BEING IN THE STUDY: There are no costs to you for participating and you will not be compensated.
BENEFITS AND RISKS There are no personal benefits or risks to participating in this study.
CONTACT INFORMATION FOR QUESTIONS AND CONCERNS: If you have concerns or questions about this study, please contact the researcher: Heather Collins, University of Kansas Medical Center, Mailstop 1050, 3901 Rainbow Boulevard, Kansas City, KS 66160. hcollins@kumc.edu or 913--588--7330.

- ❍ I work at a health sciences library associated with a medical school. By clicking this button , I voluntarily agree to participate in this online survey.
- ❍ I do not wish to participate in this survey.

Q2 What is the name of your library?

Q3 What is the name of the medical school or institution with which the library is associated?

Q4 Do librarians and/or information professionals at this institution have faculty status?
- ❍ Yes
- ❍ No
- ❍ Some, but not all librarian and information professional positions are faculty ranked
- ❍ Unique non-faculty, non-staff rank (i.e. "Community of Educators")
- ❍ Other (please describe) ____________________

Q5 In 2014, the Association of American Medical Colleges published a curriculum document, Core Entrustable Professional Activities (EPAs) for Entering Residency. Core EPAs are defined as "units of professional practice, defined as tasks or responsibilities that trainees are entrusted to perform unsupervised once they have attained sufficient specific competence." The document is available at https://members.aamc.org/eweb/upload/Core%20EPA%20Curriculum%20Dev%20Guide.pdfIs your library involved in implementing Core EPAs in the undergraduate medical curriculum at your medical school?
- ❍ Yes, librarians are working with individuals in the medical school to implement one or more Core EPAs.
- ❍ Yes, the library is working on its own project to implement one or more Core EPAs, without others at the institution.
- ❍ No, the library is not involved in implementing Core EPAs although Core EPAs are being implemented at the institution.
- ❍ No, Core EPAs are not being implemented at our institution.
- ❍ I am unsure whether Core EPAs are being implemented at our institution.

IF YES, or IF NO, THE LIBRARY IS NOT INVOLVED BUT CORE EPAS ARE BEING IMPLEMENTED, THEN PROCEED TO Q6.<br>IF NO, CORE EPAS ARE NOT BEING IMPLEMENTED OR UNSURE, PROCEED TO Q8.

Q6 Which Core EPAs are being planned and/or implemented in the undergraduate medical curriculum at your medical school? A list and description of Core EPAs is found at https://members.aamc.org/eweb/upload/Core%20EPA%20Curriculum%20Dev%20Guide.pdf

	Library is involved	Implemented or planned but library is not involved	Not being implemented or planned at the institution	Insufficient information to answer
EPA 1: Gather a history and perform a physical examination	❍	❍	❍	❍
EPA 2: Prioritize a differential diagnosis following a clinical encounter	❍	❍	❍	❍
EPA 3: Recommend and interpret common diagnostic and screening tests	❍	❍	❍	❍
EPA 4: Enter and discuss orders and prescriptions	❍	❍	❍	❍
EPA 5: Document a clinical encounter in the patient record	❍	❍	❍	❍
EPA 6: Provide an oral presentation of a clinical encounter	❍	❍	❍	❍
EPA 7: Form clinical questions and retrieve evidence to advance patient care	❍	❍	❍	❍
EPA 8: Give or receive a patient handover to	❍	❍	❍	❍

transition care responsibility				
EPA 9: Collaborate as a member of an interprofessional team	❍	❍	❍	❍
EPA 10: Recognize a patient requiring urgent or emergent care and initiate evaluation and management	❍	❍	❍	❍
EPA 11: Obtain informed consent for tests and/or procedures	❍	❍	❍	❍
EPA 12: Perform general procedures of a physician	❍	❍	❍	❍
EPA 13: Identify system failures and contribute to a culture of safety and improvement	❍	❍	❍	❍

Q7 Is your institution planning and/or implementing teaching and/or assessment of entrustable professional activities other than the Core EPAs listed above? If so, please describe.

Q8 The following is a list of the functions for Core EPA 7: "Form clinical questions and locate evidence to advance patient care." Many of these functions are often taught by librarians, even if they are not expressly working with the Core EPA framework. Which of these functions are taught by individuals from your library? Please select all of the functions which are taught by library employees as part of required activities in the undergraduate medical curriculum*, even if your library is not expressly involved in implementing Core EPAs. *Include only those activities that are a required for all medical students as part of the curriculum. Do not include activities that are taught by the library in optional workshops or electives.

	Librarians are involved in teaching this skill:			
	In the Preclinical Curriculum	In the Clinical Curriculum	In Both Preclinical and Clinical Curriculum	Not at all
Develop a well-formed, focused, pertinent clinical question	❑	❑	❑	❑
Demonstrate basic awareness and early skills in appraisal of both the sources and content of medical information using accepted criteria	❑	❑	❑	❑
Identify and demonstrate the use of information technology to access accurate and reliable online medical information	❑	❑	❑	❑
Demonstrate basic awareness and early skills in assessing applicability/generalizability of evidence and published studies to specific patients	❑	❑	❑	❑
Demonstrate curiosity, objectivity, and the use of scientific reasoning in acquisition of knowledge and application to patient care	❑	❑	❑	❑
Apply the primary findings of one’s information search to an individual patient(s)	❑	❑	❑	❑
Communicate one’s findings to the health care team (including the patient/family)	❑	❑	❑	❑

Close the loop through reflection on the process and the outcome for the patient	❑	❑	❑	❑

Q9 The following is a list of the functions for Core EPA 7 ("Form clinical questions and locate evidence to advance patient care"). Many of these functions are often assessed by librarians or information professionals, even if they are not expressly working with the Core EPA framework. Which of these functions are assessed by individuals from your library? Please select all of the functions which are assessed by library employees as part of required activities in the undergraduate medical curriculum*, even if your library is not expressly involved in implementing Core EPAs. *Include only those activities that are a required for all medical students as part of the curriculum. Do not include activities that are taught by the library in optional workshops or electives.

	Librarians are involved in assessing this skill:			
	In the Preclinical Curriculum	In the Clinical Curriculum	In Both Preclinical and Clinical Curriculum	Not at all
Develop a well-formed, focused, pertinent clinical question	❑	❑	❑	❑
Demonstrate basic awareness and early skills in appraisal of both the sources and content of medical information using accepted criteria	❑	❑	❑	❑
Identify and demonstrate the use of information technology to access accurate and reliable online medical information	❑	❑	❑	❑
Demonstrate basic awareness and early skills in assessing applicability/generalizability of evidence and published studies to specific patients	❑	❑	❑	❑
Demonstrate curiosity, objectivity, and the use of scientific reasoning in acquisition of knowledge and application to patient care	❑	❑	❑	❑
Apply the primary findings of one's information search to an individual patient(s)	❑	❑	❑	❑
Communicate one's findings to the health care team (including the	❑	❑	❑	❑

patient/family)<br>Close the loop through reflection on the process and the outcome for the patient	❑	❑	❑	❑

Q10 The following is a list of competencies that are mapped to Core EPA 13 ("Identify system failures and contribute to a culture of safety and improvement"). These competencies may be taught by librarians, even if they are not expressly working with the Core EPA framework. Which of these competencies are taught by individuals from your library? Please select all of the competencies which are taught by library employees as part of required activities in the undergraduate medical curriculum*, even if your library is not expressly involved in implementing Core EPAs. *Include only those activities that are a required for all medical students as part of the curriculum. Do not include activities that are taught by the library in optional workshops or electives.

	Librarians are involved in teaching this skill:			
	In the Preclinical Curriculum	In the Clinical Curriculum	In Both Preclinical and Clinical Curriculum	Not at all
Demonstrate an investigatory and analytic approach to clinical situations	❑	❑	❑	❑
Systematically analyze practice using quality-improvement methods and implement changes with the goal of practice improvement	❑	❑	❑	❑
Continually identify, analyze, and implement new knowledge, guidelines, standards, technologies, products, or services that have been demonstrated to improve outcomes	❑	❑	❑	❑
Communicate effectively with colleagues within one's profession or specialty, other health	❑	❑	❑	❑

professionals, and health-related agencies				
Demonstrate accountability to patients, society, and the profession	❑	❑	❑	❑
Advocate for quality patient care and optimal patient care systems	❑	❑	❑	❑
Participate in identifying system errors and implementing potential systems solutions	❑	❑	❑	❑
Develop the ability to use self-awareness of knowledge, skills, and emotional limitations to engage in appropriate help-seeking behaviors	❑	❑	❑	❑
Demonstrate trustworthiness that makes colleagues feel secure when one is responsible for the care of patients	❑	❑	❑	❑

Q11 The following is a list of competencies that are mapped to Core EPA 13 ("Identify system failures and contribute to a culture of safety and improvement"). These competencies may be assessed by librarians, even if they are not expressly working with the Core EPA framework. Which of these competencies are assessed by individuals from your library? Please select all of the competencies which are assessed by library employees as part of required activities in the undergraduate medical curriculum*, even if your library is not expressly involved in implementing Core EPAs. *Include only those activities that are a required for all medical students as part of the curriculum. Do not include activities that are taught by the library in optional workshops or electives.

	Librarians are involved in assessing this skill:			
	In the Preclinical Curriculum	In the Clinical Curriculum	In Both Preclinical and Clinical Curriculum	Not at all
Demonstrate an investigatory and analytic approach to clinical situations	❑	❑	❑	❑
Systematically analyze practice using quality-improvement methods and implement changes with the goal of practice improvement	❑	❑	❑	❑
Continually identify, analyze, and implement new knowledge, guidelines, standards, technologies, products, or services that have been demonstrated to improve outcomes	❑	❑	❑	❑
Communicate effectively with colleagues within one's profession or specialty, other health	❑	❑	❑	❑

professionals, and health-related agencies				
Demonstrate accountability to patients, society, and the profession	❑	❑	❑	❑
Advocate for quality patient care and optimal patient care systems	❑	❑	❑	❑
Participate in identifying system errors and implementing potential systems solutions	❑	❑	❑	❑
Develop the ability to use self-awareness of knowledge, skills, and emotional limitations to engage in appropriate help-seeking behaviors	❑	❑	❑	❑
Demonstrate trustworthiness that makes colleagues feel secure when one is responsible for the care of patients	❑	❑	❑	❑

Q12 Which of the following assessment methods, if any, are library employees directly using or participating in within required activities in your school's undergraduate medical curriculum? Select as many as apply.

- ❑ Written exam using free-response questions
- ❑ Written exam using multiple choice questions
- ❑ Oral exam
- ❑ OSCE (Objective Structured Clinical Examination)
- ❑ Portfolio
- ❑ Global assessment/written evaluation by faculty based on observations
- ❑ "PICO presentation:" evaluation of how student formulates a clinical question, locates evidence, appraises, and applies evidence for a specific real or hypothetical case scenario
- ❑ Librarians do not currently participate in student assessment
- ❑ Other (please describe) ____________________

Q13 Core EPAs are related to a competency-based approach in medical education. Please describe your library's involvement with competency-based medical education in general at your medical school.

QUESTIONS 14-25 ARE FOR LIBRARIES WHO <u>ARE</u> INVOLVED WITH CORE EPAS. IF YOUR LIBRARY IS NOT INVOLVED WITH CORE EPAS, PROCEED TO Q26.

Q14 Think about the way your library's involvement with Core EPAs began. Who was responsible for initiating the involvement?

- ❍ Librarians approached the medical school faculty about working on Core EPAs together.
- ❍ Medical school faculty approached librarians about working on Core EPAs together.
- ❍ Unknown
- ❍ Other (please describe) ____________________

Q15 Think about the Core EPA project(s) in which the library is involved. Which of these best describes the leadership of the project(s)?

- ❍ Leadership is controlled by the medical school faculty and does not involve the library
- ❍ Leadership is shared and involves regular library representation on task forces, education committees, etc.
- ❍ Leadership is controlled by the medical school faculty and involves the library when needed
- ❍ Leadership is controlled by the Library and does not involve medical school faculty
- ❍ Unknown
- ❍ Other (please describe) ____________________

Q16 If desired, please describe the leadership responsibility for your institution's Core EPA project(s) in greater detail.

Q17 Think about the Core EPA project(s) in which the library is involved. Which of these statements best describes how the project(s) are funded?

- The library receives funding explicitly designated for this project from the medical school
- The library receives no external funding for the project and there is no line item for the project in the library's budget.
- Unknown
- Other (please describe) ____________________

Q18 If desired, please describe the funding for your institution's Core EPA project(s) in greater detail.

Q19 Think about the Core EPA project(s) in which the library is involved. Which of these statements best describes the responsibility for teaching the knowledge and skills related to the Core EPAs in required components of the medical curriculum?

- Teaching is the sole responsibility of medical school faculty
- Teaching is a joint responsibility of the library and medical school faculty and session planning is jointly coordinated by both parties
- Teaching is a joint responsibility of the library and medical school faculty and sessions are planned and taught independently
- Teaching is the sole responsibility of librarians and/or information professionals
- Teaching does not occur
- Unknown
- Other (please describe) ____________________

Q20 If desired, please describe the teaching responsibility for your institution's Core EPA project(s) in greater detail.

Q21 Think about the Core EPA project(s) in which the library is involved. Which of these statements best describes the assessment of the Core EPAs in the required components of the medical curriculum?

- Assessment is the sole responsibility of medical school faculty
- Assessment is a joint responsibility of the library and medical school faculty and assessment planning and grading is jointly coordinated by both parties
- Assessment is joint responsibility of the library and medical school faculty and assessment planning and grading is done independently
- Assessment is the sole responsibility of librarians and/or information professionals
- Assessment does not occur/students are not evaluated
- Unknown
- Other (please describe) ____________________

Q22 If desired, please describe the assessment responsibility for your Core EPA project(s) in greater detail.

Q23 Think about information sharing related to the Core EPA project(s) in which the library is involved. Which of these statements best describes how information is shared between the medical school faculty and the library about the Core EPA project?

- Information sharing occurs regularly and results in longer-term projects beyond individual class sessions
- Information sharing pertains to specific needs or tasks related to instruction
- Information sharing is limited and conveyed only as needed
- Information sharing does not occur
- Unknown
- Other (please describe) ____________________

Q24 If desired, please describe in greater detail how information is shared between medical school faculty and the library regarding Core EPA project(s).

Q25 Think about your library's experience in working with the undergraduate medical curriculum prior to the implementation of the Core EPAs at your school. How has your library's involvement in the curriculum changed since the Core EPAs were implemented? Please list and/or describe the changes.

Q26 Please rate the significance of the following barriers or challenges regarding your library's involvement in implementing Core EPAs at your medical school.

	Not Significant	Less Significant	More Significant	Extremely significant
No orientation to Core EPAs for librarians and/or staff	❍	❍	❍	❍
Lack of time in the curriculum	❍	❍	❍	❍
Institution not currently implementing Core EPAs	❍	❍	❍	❍
No librarian/staff training or expertise in content related to Core EPAs	❍	❍	❍	❍
Lack of models from other libraries on implementing Core EPAs	❍	❍	❍	❍
Lack of resources (i.e. time, funding) in the library to implement Core EPAs	❍	❍	❍	❍
Difficulty integrating Core EPA-related content into the preclinical phase of the curriculum	❍	❍	❍	❍
Difficulty integrating Core EPA-related content into the clinical phase of the curriculum	❍	❍	❍	❍
Lack of evidence of the value of Core EPAs	❍	❍	❍	❍
Student or faculty push-	❍	❍	❍	❍

back regarding the importance of Core EPAs vs. traditional basic science or clinical content				

Q27 Please describe any other barriers or challenges that may have affected your library's involvement in implementing Core EPAs at your medical school.

Q28 The AAHSL Task Force for Competency-Based Medical Education is looking for volunteer participants for brief interviews concerning the topics covered in this survey, such as how your library is involved in implementing Core EPAs at your institution. We are also interested in talking to individuals who work in libraries that are not involved in implementing Core EPAs. If you are willing to participate in an interview, please provide your name and email address and we may contact you. If you have any questions about this survey, please contact Heather Collins, MLS, AHIP, at University of Kansas Medical Center, hcollins@kumc.edu or 913-588-7330.

Name
Email Address
